# An Established Plant Invader May Still Benefit From Increasing Genetic Diversity—Insights From Artificial Populations in a Common Garden Experiment

**DOI:** 10.1002/ece3.70963

**Published:** 2025-02-14

**Authors:** L. Y. Watermann, W. Durka, A. Erfmeier

**Affiliations:** ^1^ Kiel University, Institute for Ecosystem Research/Geobotany Kiel Germany; ^2^ Department of Community Ecology Helmholtz Centre for Environmental Research – UFZ Halle (Saale) Germany; ^3^ German Centre for Integrative Biodiversity Research (iDiv) Halle‐Jena‐Leipzig Leipzig Germany

## Abstract

Genetic diversity and competitive ability, though extensively studied in the context of biological invasions, are still poorly understood in their relative importance, especially when shifting the perspective from an individual plant's phenotype to overall population performance. Most approaches addressing the role of genetic diversity involve the comparison of standing genetic variation in field populations combined with experimental treatments on individual plants. Composing predefined mixtures of populations to manipulate genetic diversity would be an experimental approach to test for direct effects on population performance. We determined pairwise genetic distances among 16 invasive and 22 native populations of *Jacobaea vulgaris* GAERTN. using single nucleotide polymorphisms (SNP). Based on this information, we created each 15 de‐novo populations with different levels of genetic diversity for both origins. These de‐novo populations were subjected to three levels of decreasing microsite availability by using a matrix of either 0, 5, or 10 individuals of *Festuca rubra* . We monitored population performance continuously throughout two growing seasons to study effects of interactions between origin, microsite availability, and genetic diversity with (generalized) linear effects models. This allowed us to uncover whether the relative importance of those factors varies with the life‐stage of this biennial species. We found no ambiguous patterns on the hypothesized beneficial effect of genetic diversity for *J. vulgaris* populations. Native populations tended to respond negatively to increasing genetic diversity, especially under more favorable site conditions, but this was not a persistent pattern and was only evident through continuous monitoring. Invasive populations could benefit from increasing genetic diversity during early establishment, but not in interaction with restricted microsite availability. Our results do suggest that genetic variation supports population establishment and performance under certain environmental conditions. Therefore, for recommendations in nature conservation, efforts should still aim at limiting propagule addition in already invaded areas, even in well‐established invasive species.

## Graphical abstract

Genetic diversity and competitive ability, though extensively studied in the context of biological invasions, are still poorly understood in their relative importance, especially when shifting the perspective from an individual plant's phenotype to overall population performance. We created each 15 de‐novo populations with different levels of genetic diversity for native and invasive origins of the highly successful invasive *Jacobaea vulgaris* . While native populations' performance was mostly decoupled from genetic diversity, invasive populations could benefit from increasing genetic diversity during early establishment, supporting aims to limit propagule addition even for well‐established invasive species.

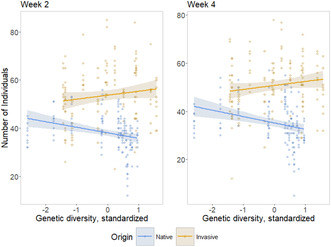

## 1 Introduction

Biological invasions are an immanent aspect of past and present anthropogenic impacts (Bellini et al. 2022; Hui and Richardson 2017) and, consequently, ongoing global change. Increasing human mobility allowed invasive alien species to overcome formerly insurmountable (geographical) barriers and many invasive species have shown copious negative reverberations (Kumar Rai and Singh 2020). Among these effects are a loss of biodiversity (Gaertner et al. 2009; Linders et al. 2019; McGeoch et al. 2010) and impairment of ecosystem services (Pejchar and Mooney 2009). Management of invasive species is difficult and costly (Diagne et al. 2021; Haubrock et al. 2021; Hulme 2020) and rapid detection measures, allowing to interject earlier in the invasion process, have just lately become more feasible (Larson et al. 2020). While prevention and early intervention have the best cost–benefit ratio (e.g., Mack et al. 2000), being able to identify high‐risk alien species and scenarios to allow efficient use of available resources (e.g., Ahmed et al. 2022) requires an in‐depth understanding of the mechanisms at play.

Inherent features of invasive populations, such as their genetic architecture, may fundamentally impact the invasion process and determine the success or failure of a population's establishment in a novel habitat. Invasion success was shown to be positively affected by high intraspecific genotypic diversity specifically (Forsman 2014; Lavergne and Molofsky 2007; but see Rius and Darling 2014). If intraspecific genetic diversity is high, the probability increases for the presence of genotypes well‐suited for the novel environment (Lachmuth et al. 2010; Schlaepfer et al. 2010). In addition, genetic diversity is also a precondition for selection. In the context of biological invasions, the absence of “invasion‐bottlenecks” as well as within (invasive) range‐admixture resulting in high levels of genetic diversity were shown to aid the invasion process (e.g., Hahn and Rieseberg 2017; Keller and Taylor 2010; Vicente et al. 2021). Specifically, intraspecific diversity might act as a pool for selection in the novel environment, just as proposed for biodiversity under the insurance theory (Loreau et al. 2021; Wright et al. 2021). High levels of genetic diversity thus reinforce adaptive processes, enabling invasive populations to overcome limiting environmental conditions and avoid (local) extinction via evolutionary rescue (Ashander et al. 2016; Carlson et al. 2014; Erfmeier 2013; Feiner et al. 2021).

At the same time, environmental factors that prevail at the initial establishment site determine a community's invasibility and thereby an invasive populations' fate. Establishment of invasive populations is particularly promoted by anthropogenic disturbances (Bellini et al. 2022; Kumar Rai and Singh 2020), that may even aid in bypassing a community's resistance to biological invasions (Belote et al. 2008). For example, as factors associated with disturbance, increased microsite availability (Davis et al. 2000) or reduced competition (Kneitel and Perrault 2006) support seedling establishment. Habitats characterized by high levels of disturbances can subsequently act as starting points for further expansion into landscapes (e.g., Haider et al. 2018; Hood and Naiman 2000; Turner et al. 2021). To become successful, invasive populations must therefore first be able to occupy these disturbed habitats, while subsequently exhibiting sufficient competitive ability to successfully invade areas beyond those entry points characterized by disturbance.

*Jacobaea vulgaris* gaertn. constitutes a suitable model species to address the importance of genetic diversity in relation to microsite availability during invasion. The species is native to Eurasia and was introduced to Australia, New Zealand, and North America during the last century (Harper and Wood 1957). In the native range, *J. vulgaris* populations revealed no signs of isolation by distance within Northern Germany, as evidenced in a genetic marker study, which was corroborated by the absence of chemotype differentiation (Jung et al. 2020). Furthermore, in the invasive range(s), including Australia, New Zealand, and North America, low genetic differentiation was found, implying either multiple introduction events or admixture following the initial introduction (Doorduin et al. 2010). In its native range, the species is known as a typical ruderal species and weed in extensively used grasslands and is often found in disturbed habitats (Cameron 1935; Harper and Wood 1957). Its population size usually decreases in later successional stages (van de Voorde et al. 2012). In this species, high intraspecific diversity might be one of the drivers of successful invasion in various novel habitats. In particular, in habitats with low levels of resources, invasibility might particularly be increased when genetic diversity in founder populations is high.

To study how both genetic diversity and microsite availability (as one main consequence of disturbance) affect the invasion success of species, we conducted a common garden experiment with native and invasive origins, different levels of genetic variation, and of microsite availability for establishment. We manipulated genetic diversity, using seeds of 22 native and 16 invasive source populations to create experimental de‐novo populations. Following a gradient of increasing genetic diversity, 15 native and invasive de novo populations each were subjected to three levels of microsite availability. Our empirical approach specifically addresses genetic diversity on the population level. Here, we explicitly move the focus away from sampling single genotypes as replicates of populations and shift towards considering the composition of populations as study units.

With this set‐up, we addressed the hypothesis that (I) in invasive populations, increasing levels of genetic diversity promote establishment success more than in native populations, while (II) increased levels of genetic diversity generally mitigate the negative impact of low microsite availability. Lastly, (III) we expect that the impact of increased genetic diversity at low microsite availability is more important for invasive than for native origins.

## 2 Material and Methods

For the common garden experiment, both seeds and leaves were collected from native and invasive source populations of *J. vulgaris* (Table S1; Figure S2). Leaf material was used to determine genetic differentiation between source populations within the two ranges (Figure S3). Pairwise *F*_ST_ values between populations were used to create artificial de‐novo populations differing in genetic variation (Figures S7 and S8: Mixture selection). Experimental units were prepared with different densities of *Festuca rubra* that served as a matrix organism. The de‐novo populations were then subjected to these pre‐planted experimental units. The performance of emerging seedlings and growing plants was monitored over 45 weeks (i.e., the entire life‐cycle of the biennial model species) to assess how genetic diversity and microsite availability impact a population's performance depending on the origin of the seeds.

### 2.1 Study Species

The herbaceous Asteraceae *J. vulgaris* gaertn. (syn. *Senecio jacobaea* L.) is nearly globally distributed. It originates from Eurasia and was introduced to the North American East Coast in the mid‐19th century, to Australia by the end of the 19th century (Schmidl 1972), and was first recorded at the North American West Coast at the beginning of the last century (Isaacson 1973). In its native range, *J. vulgaris* has received increasing attention as a “problematic weed” with massive expansion and invasive‐like patterns in various regions during the last decades (Jung et al. 2020; Neumann et al. 2015; Suter et al. 2007). For Switzerland, Bosshard et al. (2003) pinpoint this increase in abundance around the turn of the millennium. The species is oftentimes considered problematic, especially by landowners and livestock farmers due to its toxic compounds (e.g., Gottschalk 2018; Gottschalk et al. 2020).

*Jacobaea vulgaris* is a typical ruderal species, often observed at road verges and early successional sites. It depends on open soil for successful establishment (Cameron 1935) and exhibits a predominantly biennial life‐cycle (Wardle 1987). In the first growing season, individuals form a rosette and develop a sprout in the following year. Both the size of the first‐year rosette (van der Meijden and van der Waals‐Kooi 1979; Wesselingh and Klinkhamer 1996), as well as nutrient availability (Prins et al. 1990) determine whether an individual plant will flower in the subsequent year. *J. vulgaris* is outcrossing and is pollinated by several generalist pollinators (Andersson 2001). When flowering, *J. vulgaris* individuals regularly produce up to 30,000 achenes (Schmidl 1972; van der Meijden and van der Waals‐Kooi 1979) most of which germinate directly in autumn of the same year (Harper and Wood 1957).

### 2.2 Sampling Design

In late summer 2018, seed and leaf material of *J. vulgaris* was collected from 22 native (Central and Northern Europa) and 16 invasive (Pacific Northwest) populations (for exact locations Table S1; Figure S2). Within the Pacific Northwest, sampling was designed to include as much of the species' range as possible. In the species' native range, the sampling spanned an area that is characterized by a noticeable increase in abundance over the last two decades (according to information from local nature conservation authorities and landowners), mostly involving large areas in Northern Germany. In both ranges, populations were sampled that vary in their population characteristics (size, extent, density, etc.) and cover different habitats (ruderal location, managed grassland like meadows, pastures, etc.). Within each of these source populations, we aimed to sample 20 individuals (due to requiring both enough non‐senescent leaves and viable seeds from the same maternal plant, this wasn't always possible) along a transect to capture the genetic diversity of a population as representatively as possible. Plants were sampled at least 2 m apart. Sampled achenes (hereinafter “seeds”) were stored separately by individual. All collected material was kept dry in paper bags until further use.

### 2.3 Genetic Analysis

Genetic variation of source populations was quantified to serve as a basis to create artificial experimental populations (de‐novo populations) with different levels of genetic variation. We adopted a population pool approach (Futschik and Schlötterer 2010) for the analysis of genomic variation. Similar to Conrady et al. (2022) this approach allows a focus on genetic variation at the population level exclusively. Therefore, for each source population, the same amount of leaf material was pooled from each of the 20 maternal plants. We used a reduced representation sequencing approach for single‐nucleotide polymorphism (SNP) detection following the ddRAD protocol of Peterson et al. (2012) (for further details see Appendix S4: Genetic analysis). Sequence reads were demultiplexed with process_radtags from Stacks 2.0 (Catchen et al. 2013) and assembly of a de‐novo reference genome, SNP detection, and genotyping was done with dDocent 2.6.0 (Puritz et al. 2014). Finally, for preliminary analysis to be able to create the de‐novo populations, SNPs were filtered following O'Leary et al. (O'Leary et al. 2018). Indels were removed, and only biallelic loci were kept. Furthermore, only loci with less than 66% missing values, a minor allele count of three and a minimum read depth of 20 were retained, removing all pools with more than 75% missing data and finally retaining only one SNP per contig. For the final analysis, used only for statistical analysis of the final results, only loci with less than 50% missing values and a minimum read depth of 40 were retained. As a measure of genetic diversity of source and de‐novo populations, we calculated unbiased expected heterozygosity (*H*_e_) from allele frequencies, that is, the number of read counts of the reference allele divided by the total read count, after Nei and Roychoudhury (1974) and averaged across loci. Pairwise genetic distances (*F*_ST_) among source populations were calculated using the package poolfstat (Gautier et al. 2022) in R (R Core Team 2019) (see Figure S5 for full *F*_ST_ table for both origins). Total genetic diversity was higher in the native than in the invasive range (*H*_T_ = 0.280 and 0.273, respectively, calculated by summing up read counts across samples). However, mean *H*_e_ of the source populations was comparable between origins (0.203 ± 0.0123 for invasive and 0.206 ± 0.0102 for native origins), while differentiation among populations (*F*_ST_) was significantly lower (Welch's *t*‐test *p* = < 0.001) in the invasive range (0.0436 ± 0.0276) than in the native range (0.0578 ± 0.028). We found significant isolation‐by‐distance in both origins by Mantel test using vegan 2.6–4 (Oksanen et al. 2024) (Figure S6 for visualization).

### 2.4 Experimental de‐Novo Populations

Experimental de‐novo populations were compiled separately for native and invasive origins. For creating de‐novo populations, seeds from each of the five sampled source populations were combined. Along the full range of possible values of genetic diversity of population mixes for both native and invasive origins, as predicted based on the mean pairwise *F*_ST_‐value among the five mixed populations, 15 mixes were randomly selected (Figure S7). Random selection followed a stratified design to ensure that the full gradient per region of origin was also covered (for details of the selection procedure see Appendix S8: Mixture selection). Ultimately, the diversity levels of the artificial de‐novo populations, calculated by summing up read counts across the mixed source populations, ranged from *H*_e_ = 0.238 to 0.280 for native and from 0.259 to 0.268 for invasive origins.

For the experiment, new seed sample mixtures representing the de‐novo populations were created by assembling seed mixtures containing equal proportions of seeds from all maternal plants of the respective five source populations each, that is, each seed sample mixture comprised 100 seeds of which 20 originated from each of the five source populations. Seed quality was checked by visual inspection (size and color), and only viable seeds were included. Nine exact replicates were separately prepared per de‐novo population to be assigned to different treatments in the common garden experiment (see below). For each final de‐novo population seed sample, an image of the final 100 seeds was taken with a scanner (Expression 11000XL, Seiko Epson Corporation) and seed size (average projected area per seed) was analyzed using WinFolia (V.2015b 32 bit, Regents Instruments Canada Inc.). The nine replicate samples were randomly assigned to experimental treatments in the common garden experiment.

### 2.5 Common Garden Experiment

For the common garden experiment, treatments of manipulated genetic diversity and microsite availability were realized in a fully crossed design. While the manipulation of genetic diversity was implemented with experimental de‐novo populations, microsite availability was implemented with three levels of open soil realized by planting a matrix organism in different numbers. As a suitable matrix organism, *Festuca rubra agg*. L. was chosen as it is widely distributed in the northern hemisphere. Levels of high, medium, and low microsite availability were created by planting either no (zero), 5 or 10 *F. rubra* seedlings, respectively. A similar set‐up was used by Erfmeier et al. (2013) with *Festuca rupicola*. Each experimental unit in the present experiment was prepared as a 7.5 L planting container (7.5 L SMH3, Soparco) lined at the bottom with chemical‐free weed fleece (Gardol Premium Unkrautvlies, DuPont de Nemours (Deutschland) GmbH) and filled with a 40:60%_v_ substrate mix of sand provided by the Botanical Garden Kiel (Germany) and unfertilized potting soil (F.‐E. Typ Nullerde, HAWITA Gruppe GmbH). To each container (henceforth addressed as experimental units), 3 g/L slow‐release fertilizer (Osmocote Exact Hi. End 12‐14 M, ICL Specialty Fertilizers) was added to ensure a continuous supply of nutrients during the runtime of the experiment. Seeds of *F. rubra* were provided by the Botanical Garden Halle (Saale) (Germany). They were sown in sowing trays 3 weeks prior to the experimental starting date. After 2 weeks, seedlings were transferred to the experimental units and survival was monitored for another week, replacing dead seedlings.

Seed samples for establishing de‐novo populations were added 1 week after planting the *Festuca‐*matrix (this also marked the time after which no more *Festuca* seedlings were replaced). To prevent drying out of seeds and to protect them from external disturbance, seeds were covered with a thin layer of substrate (about 3–5 mm). After seed addition, all experimental units were kept in the greenhouse for another week and kept consistently moist by daily watering.

In accordance with the primary window for germination under field conditions (Harper and Wood 1957) the experimental units were transferred to an outside area by Mid‐August 2019. The total experiment comprised 270 experimental units (15 different de‐novo populations with varying genetic diversity per 2 origins in 3 microsite levels with 3 replicates each) placed in a random order outside in the Botanical Garden of Kiel University (Germany). The area was specifically chosen as no wild populations of *J. vulgaris* were within proximity. Eight additional experimental units only containing substrate served as control and confirmed no influx of *J. vulgaris* propagules from the outside during the experiment. The experimental set‐up was only once turned 180° during the second growing season for control.

The experimental area was fenced to prevent damage by larger herbivores (mainly rabbits). Caterpillars and slugs as well as weeds were continuously removed while rodents were controlled using traps. During the first 4 weeks, containers were watered up to twice daily to ensure seed germination. Watering was picked up in accordance with weather conditions in their interaction with plant growth late in the second growing season in 2020 when the amount of biomass in the experimental units required watering up to twice daily.

### 2.6 Monitoring and Data Collection

Monitoring was carried out continuously during the experiment to capture all growth phases and only paused during winter months. At each monitoring date, all vegetative individuals were counted and assigned to one of 10 size classes (assigned both by life‐stages and size) sensu Erfmeier et al. (2013):

Class I (vegetative; cotyledons only), Class II (vegetative; longest leaf < 1 cm), Class III (vegetative; longest leaf 1 ≥ 4 cm), Class IV (vegetative; longest leaf 4 ≥ 8 cm), Class V (vegetative; longest leaf 8 ≥ 12 cm), Class VI (vegetative; longest leaf ≥ 12 cm), Class VII (bolting), Class VIII (bolting with inflorescences formed), Class IX (bolting and fully flowering), Class X (bolting and seed set start).

As a measure for relative growth, the size‐class ratio per experimental unit was calculated as follows:

$$\text{Size class ratio}=\ln\frac{\text{Number of}\;\left(\text{Class}\;\mathrm{II}+\text{Class}\;\mathrm{III}+\text{Class}\;\mathrm{IV}+\text{Class}\;V+\text{Class}\;\mathrm{VI}\right)+1}{\text{Number of Class}\;I+1}$$

Inflorescences were removed when seed development started to avoid spreading *J. vulgaris* seeds in the surrounding area (those individuals were still classified as Class X).

The experiment ran for 45 weeks (from August 2019 to June 2020), that is, it comprised life phases in two consecutive growing seasons, including the winter‐dormancy period. After 45 weeks, reproductive and vegetative biomass were harvested separately, dried for at least 48 h in a drying oven at 65°C with subsequent dry‐weight determination.

### 2.7 Statistical Analyses

Statistical analyses were carried out using R (v4.3.1) (R Core Team 2019). All plots were created using ggplot2 (v3.4.3) (Wickham 2016). For the genetic diversity of the de‐novo populations, we used their calculated *H*_e_ values. Since the value ranges differed substantially (0.2377–0.2795 for native and 0.2593–0.2676 for invasive origins), we standardized the *H*_e_ value within each origin (native and invasive) using z‐transformation sensu Hock et al. (2019).

In all models, genetic diversity, microsite availability, and origin were used as fixed effects. For size parameters, a linear model was fitted for each monitoring date. After checking residuals, we used a generalized, zero‐inflated model (family = ziGamma), where microsite availability was addressed as the cause of zero inflation (glmmTMB v1.1.7) (Brooks et al. 2017) for size parameters for Weeks 11 and 16 and all harvest data variables. The number of individuals during continuous monitoring was assessed with a generalized linear model using the “quasipoisson” family. To account for maternal effects, the average projected area of seeds was incorporated as a covariate until Week 4. For biomass variables (vegetative and total biomass) one sample had to be excluded. Estimates and significance of trends were determined for the corresponding subset of the data with the general models described above (without the predictor used to separate the data). For estimate calculation, however, zero‐inflation models were only used for vegetative biomass, separation by microsite level meant that the general zero‐inflation term was used (ziformula = ~1).

Model validation (residual check) was done using the DHARMA package (v0.4.6) (Hartig 2021) checking both the Q‐Q‐plot and the residual versus prediction‐plot.

## 3 Results

Germination was successful in all experimental units. The highest number of individuals at a single monitoring was 91 within one experimental unit in Week 3. The number of individuals increased continuously during the first weeks and then decreased until the end of the first growing season. In the second season, successful bolting and flowering were observed. There was no significant effect of seed size (average projected area), neither for size nor for the number of individuals (Tables 1 and 2).

**TABLE 1:** Number of individuals. Number of individuals observed per experimental unit. ANOVA‐results from the generalized linear model.

	Week 1			Week 2			Week 4		
	Chi square	Df	*p*	Chi square	Df	*p*	Chi square	Df	*p*
GD	0.23386	1	0.6287	4.062	1	**0.044**	3.175	1	0.075
MSA	1.57268	2	0.4555	0.015	2	0.992	0.648	2	0.723
Origin	0.05	1	0.8231	58.917	1	**< 0.001**	62.372	1	**< 0.001**
Seed Size	0.13084	1	0.7176	0.001	1	0.981	0.629	1	0.428
GD × MSA	0.62606	2	0.7312	0.728	2	0.695	1.771	2	0.413
GD × Origin	0.2164	1	0.6418	4.28	1	**0.039**	6.96	1	**0.008**
MSA × Origin	1.48942	2	0.4749	3.91	2	0.142	5.967	2	0.051
GD × MSA × Origin	1.20823	2	0.5466	1.921	2	0.383	2.195	2	0.334

	Week 11			Week 16			Week 40		
	Chi square	Df	*p*	Chi square	Df	*p*	Chi square	Df	*p*
GD	0.0295	1	0.864	0.0132	1	0.909	0.222	1	0.638
MSA	8.6951	2	**0.013**	16.3036	2	**< 0.001**	219.163	2	**< 0.001**
Origin	25.2522	1	**< 0.001**	5.9744	1	**0.015**	0.976	1	0.323
GD × MSA	6.341	2	**0.042**	2.1613	2	0.339	5.494	2	0.064
GD × Origin	0.137	1	0.711	0.0000	1	1.000	0.313	1	0.576
MSA × Origin	4.968	2	0.083	1.6932	2	0.429	3.378	2	0.185
GD × MSA × Origin	6.3219	2	**0.042**	1.5404	2	0.463	6.502	2	**0.039**

*Note:* Significant effects (*p* < 0.05) are printed in bold.

Abbreviations: GD, genetic diversity; MSA, microsite availability.

**TABLE 2:** Size of the individuals. For Weeks 1–4, the size class ratio was calculated. Afterwards, size refers to the rosette size ratio (threshold of 8 cm). ANOVA‐results from the (generalized) linear model.

	Week 1				Week 2				Week 4			
	Sum square	Df	*F*	*p*	Sum square	Df	*F*	*p*	Sum square	Df	*F*	*p*
GD	0.0434	1	0.3596	0.549	0.539	1	1.4662	0.227	0.045	1	0.1181	0.731
MSA	0.318	2	1.3162	0.270	0.494	2	0.6715	0.512	0.356	2	0.4673	0.627
Origin	0.0073	1	0.0604	0.806	11.555	1	31.4344	**< 0.001**	0.242	1	0.637	0.426
Seed Size	0.296	1	0.2448	0.621	1.335	1	3.6316	0.058	0.044	1	0.1145	0.735
GD × MSA	0.1028	2	0.4254	0.654	0.113	2	0.1537	0.858	0.426	2	0.5594	0.572
GD × Origin	0.186	1	0.1543	0.695	0.007	1	0.019	0.891	0.553	1	1.4519	0.229
MSA × Origin	0.1881	2	0.7786	0.460	0.405	2	0.5512	0.577	0.694	2	0.9117	0.403
GD × MSA × Origin	0.177	2	0.7328	0.482	0.503	2	0.6847	0.505	0.724	2	0.9518	0.387

	Week 11^1^			Week 16^1^			Week 40			
	Chi square	Df	*p*	Chi square	Df	*p*	Sum square	Df	*F*	*p*
GD	0.0214	1	0.884	0.0602	1	0.8061	2.2	1	0.2241	0.636
MSA	9.8527	2	**0.007**	3.9261	2	0.1404	2777.6	2	141.508	**< 0.001**
Origin	2.0066	1	0.156	1.7707	1	0.1833	37.8	1	3.8471	0.509
GD × MSA	1.8863	2	0.389	0.2059	2	0.9022	31.1	2	1.5834	0.207
GD × Origin	1.053	1	0.305	0.3222	1	0.5703	12.7	1	1.2931	0.257
MSA × Origin	3.9837	2	0.136	0.4052	2	0.8166	59.9	2	3.0531	**0.049**
GD × MSA × Origin	0.1982	2	0.906	0.9193	2	0.6315	31.5	2	1.6024	0.203

*Note:* Seed Size measured as average projected area of seeds (determined for each sample seperately). 1 Model with zero‐inflation to account for zero‐inflation caused by MSA. Significant effects (*p* < 0.05) are printed in bold.

Abbreviations: GD, genetic diversity; MSA, microsite availability.

**FIGURE 1:**
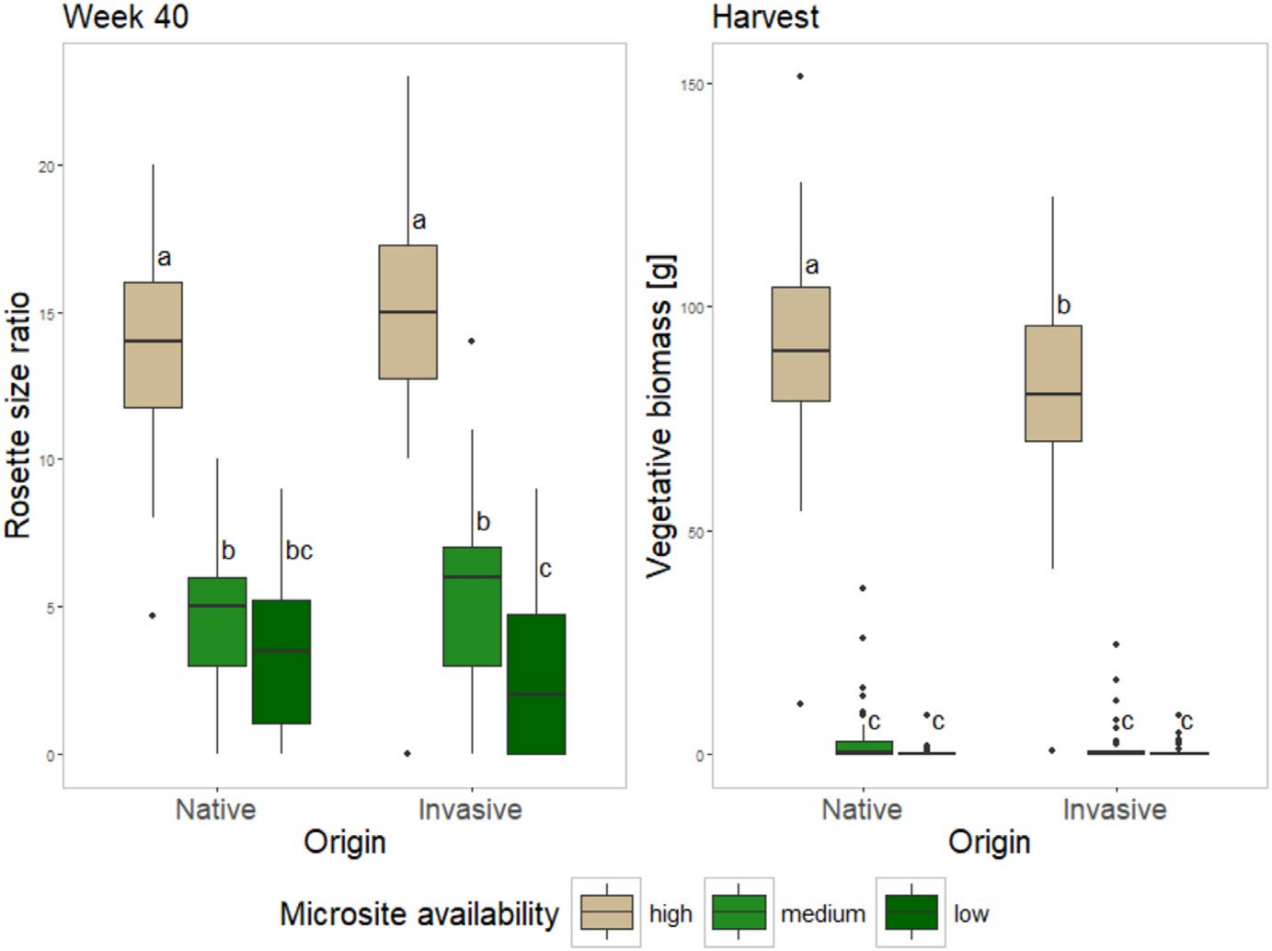
Microsite level × origin effects. Rosette size ratio (indicating relative proportion of rosettes larger than 8 cm compared to smaller rosettes) after Week 40 on the left. Vegetative biomass at the harvest on the right. The three left boxplots depict performance of invasive de‐novo populations, invasive origins are depicted by the three right boxplots. Levels of microsite availability are given in decreasing order left (high level) to the right (low level). Tukey post hoc test was used to find individual differences.

**Origin** significantly affected the number of individuals growing in each experimental unit between Week 2 and 16, with invasive origins consistently outperforming native populations (Table 1). The size of the individuals, however, did not differ between origins, except in Week 2 when native origins had a higher proportion of developed individuals than seedlings compared to invasives indicating an earlier germination (Table 2). **Genetic diversity** had no effect on plant size and affected the number of individuals only in Week 2. **Microsite availability**, in contrast, consistently affected both number and size starting from Week 11 (note that for size at Week 16, microsite was a cause for zero‐inflation with both densities significantly deviating from the control). All responses addressed during harvest were significantly impacted by microsite availability as well (Table 3). The interaction between **Microsite availability** and **Origin** significantly affected the rosette size ratio in Week 40, as well as vegetative and total biomass at harvest (Tables 1 and 3). Rosette size ratio was different between all microsite availabilities for invasive origins (Figure 1). For native origins, only the high level of microsite availability led to significantly larger individuals, whereas there was no additional difference between medium and low levels of microsite availability. At the lowest levels of microsite availability, there was no difference in the response between the origins. At harvest, invasive de‐novo populations also had more vegetative biomass at high levels of microsite availability compared to the native origins. However, the increase in vegetative biomass from low and medium levels to high levels of microsite availability was much steeper for native origins compared to invasive ones.

**TABLE 3:** Harvest data. Results of harvest monitoring. ANOVA results from the generalized linear model.

	Number of individuals			Life stage		
	Chi square	Df	*p*	Chi square	Df	*p*
GD	0.0762	1	0.783	0.2772	1	0.599
MSA	150.8583	2	**< 0.001**	100.2105	2	**< 0.001**
Origin	0.0031	1	0.956	0.6251	1	0.430
GD × MSA	2.6515	2	0.266	3.5212	2	0.172
GD × Origin	0.2217	1	0.638	4.3257	1	**0.038**
MSA × Origin	1.2706	2	0.530	0.7735	2	0.679
GD × MSA × Origin	2.1346	2	0.344	8.055	2	**0.018**

	Vegetative biomass [g]			Total biomass [g]		
	Chi square	Df	*p*	Chi square	Df	*p*
GD	0.0199	1	0.888	0.0246	1	0.875
MSA	397.1764	2	**< 0.001**	225.068	2	**< 0.001**
Origin	2.0782	1	0.149	0.5499	1	0.458
GD × MSA	9.4916	2	**0.002**	1.8652	2	0.169
GD × Origin	0.0689	1	0.793	0.02	1	0.888
MSA × Origin	13.6756	2	**< 0.001**	7.8469	2	**0.005**
GD × MSA × Origin	1.2456	2	0.264	1.0728	2	0.300

*Note:* Significant effects (*p* < 0.05) are printed in bold.

Abbreviations: GD, genetic diversity; MSA, microsite availability.

**FIGURE 2:**
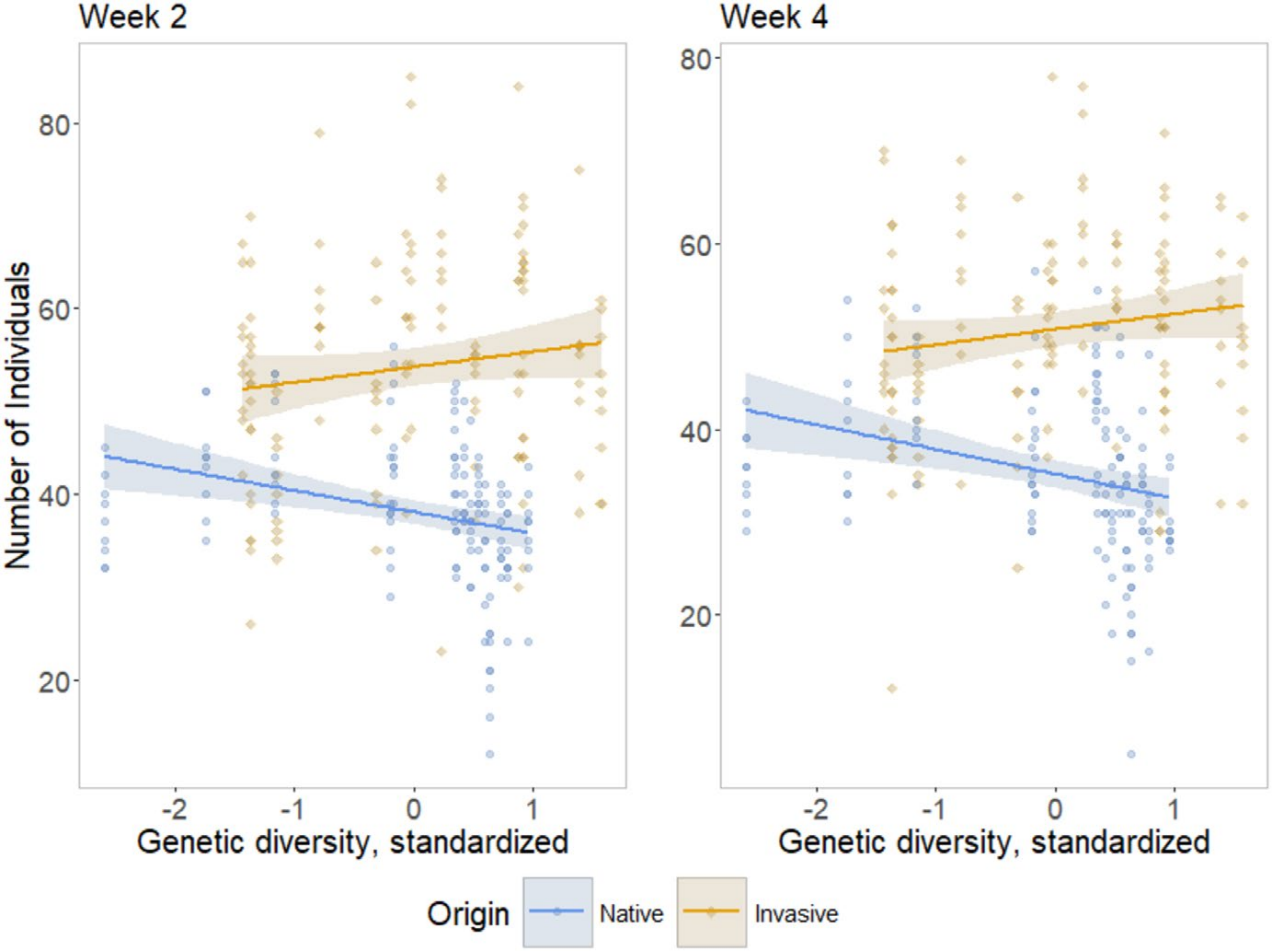
Genetic diversity × origin effects. Number of individuals in each experimental unit at Week 2 (left) and Week 4 (right). Square (blue) dots represent native populations. Colored areas indicate the 95% confidence interval predicted from linear model. Please consider: Genetic diversity was standardized for each origin separately.

**Genetic diversity** showed significant interaction effects with **Origin** concerning the number of individuals at week 2 and 4 (Table 1, Figure 2) as well as the relative proportion of generative to vegetative individuals (life‐stage) at the end of the experiment (Table 3). While native de‐novo populations showed a significant negative relationship between the number of individuals and genetic diversity in Week 2 (*p* = 0.018, Estimate = −0.0684), in week 4 only invasive de‐novo populations showed a significant (positive) relationship (*p* = 0.047, Estimate = 0.0608). At harvest, invasive de‐novo populations had a relatively smaller number of generative to vegetative individuals with increasing genetic diversity (*p* = 0.015, Estimate = −0,2531).

**FIGURE 3:**
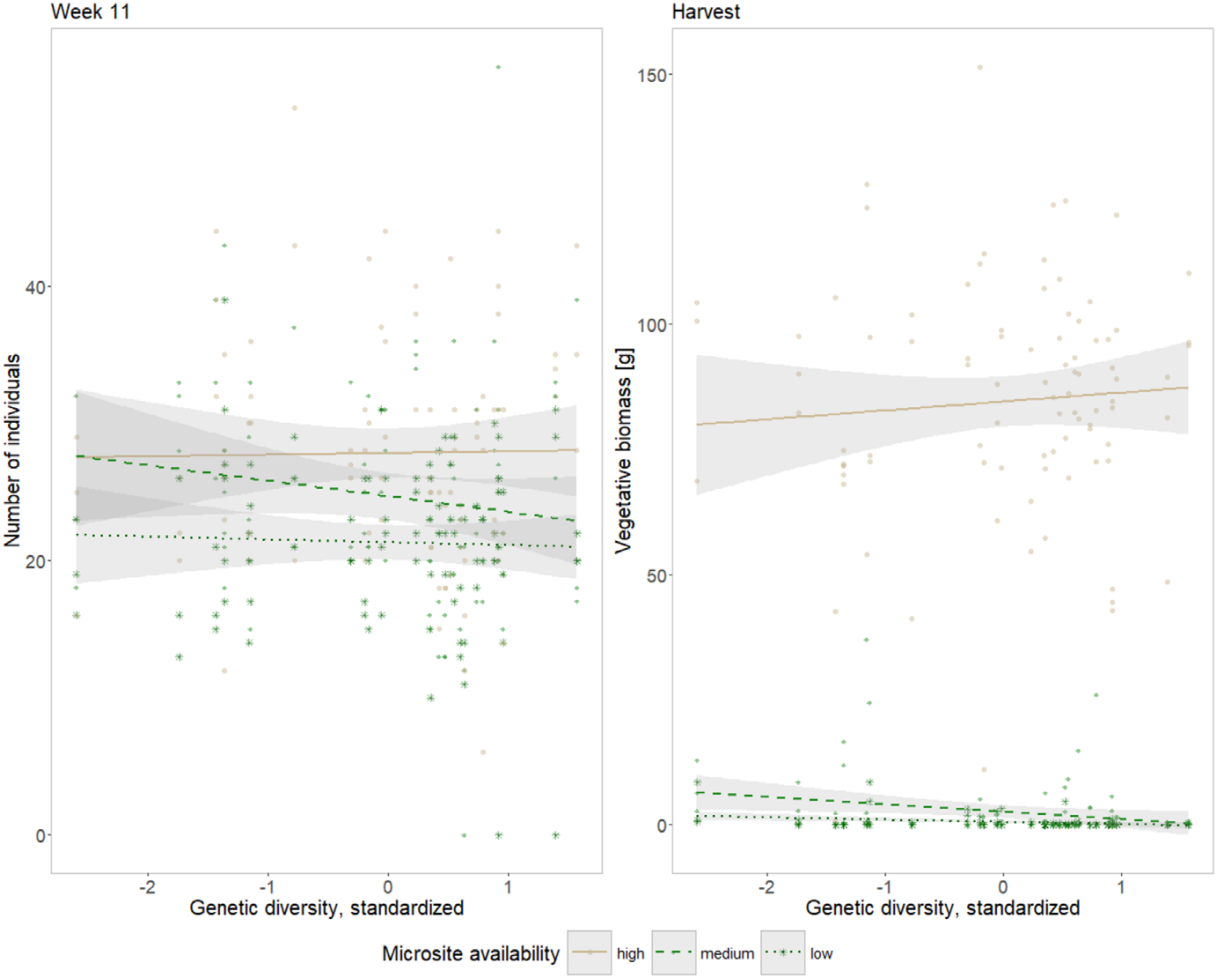
Genetic diversity × microsite level effects. Number of individuals in each experimental unit (left) and vegetative biomass as evaluated at harvest (right). Levels of microsite availability are given in decreasing order left (high level) to the right (low level). Gray areas indicate the 95% confidence interval predicted from linear model. Please consider: Genetic diversity was standardized for each origin separately.

**Microsite availability and genetic diversity** displayed strong interaction effects in the number of individuals in Week 11 as well as in the harvested vegetative biomass (Tables 1 and 3; Figure 3). At Week 11, the number of individuals was negatively affected by genetic diversity under medium microsite availability (*p* = 0.005, Estimate = −0.1229), while there was no significant relationship under high or low microsite availability. The vegetative biomass was negatively affected by increasing genetic diversity under low microsite availability (*p* = 0.003, Estimate = −0.7450), while the negative effects under medium and high microsite availability (Estimate_(medium)_ = −0.2713 and Estimate_(high)_ = −0.0090) were not significant (*p*_(medium)_ = 0.2722 and *p*_(high)_ = 0.8829).

**FIGURE 4:**
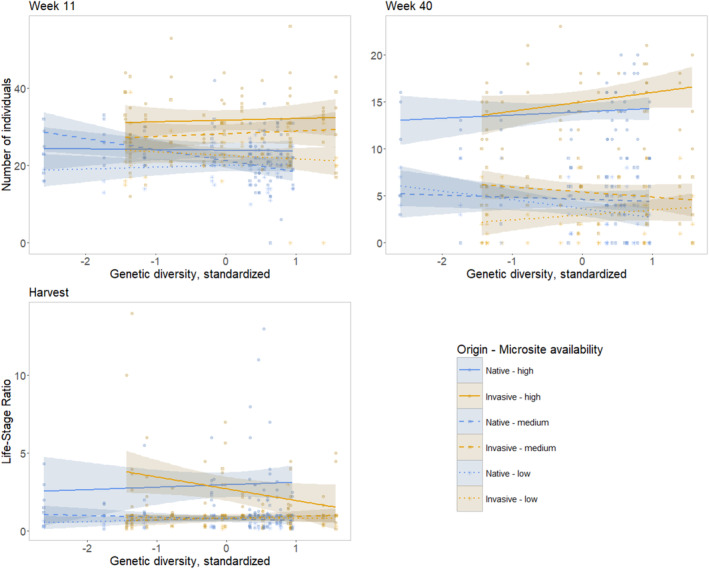
Origin × Microsite availability × genetic diversity effects. Number of individuals at Week 11 (top left) and Week 40 (top right) and proportion of generative to vegetative individuals at harvest (bottom left). Levels of microsite availability are given in decreasing order from left to right: High level = solid line, medium level = dashed line, low level = dotted line. Colored areas indicate the 95% confidence interval predicted from linear model. Please consider: Genetic diversity was standardized for each origin separately.

Three response traits showed a significant three‐way interaction between **origin**, **genetic diversity**, and **microsite availability** at individual monitoring dates (Figure 4). In native de‐novo populations, the number of individuals in Week 11 was significantly negatively affected by genetic diversity under medium microsite availability (*p* = 0.0039, Estimate = −0.1229), whereas there was no significant effect for the other combinations. After 40 weeks, native de‐novo populations growing in low microsite availability exhibited a negative relationship between the number of individuals and increasing genetic diversity (*p* = 0.0231, Estimate = −0.2212) while the positive trend in high microsite availability was not significant in both origins. The life‐stage ratio showed a significant negative relationship in invasive de‐novo populations in high microsite availability with increasing genetic diversity (*p* = 0.0498, Estimate = −0.7526).

## 4 Discussion

### 4.1 Initial Establishment Is Affected by Origin and Genetic Diversity

Independent of other factors examined in our experiment, invasive origins exhibited a higher initial establishment success than native ones, as shown by the main effects in the variables number and size of individuals. These early observations are in line with previous studies. Higher germination success has often been observed for invasive origins of species (e.g., Beckmann et al. 2011; Hock et al. 2015; Xu et al. 2019; Zhou and He 2020), which was also the case with our source populations (unpublished data). However, this effect did not translate into generally higher performance in the present experiment. This is in contrast to previous studies, which found that invasive individuals of *J. vulgaris* grow larger (Joshi and Vrieling 2005) and accumulate more biomass (Lin et al. 2019) under non‐competitive conditions as well as in a common garden experiment in the native range (Stastny et al. 2005). However, since these experiments focused on single individuals rather than populations, findings may not be directly transferable. Individuals in our de‐novo populations were subjected to a high level of intraspecific competition, which may have limited performance potential both on an individual and a population level, especially at later developmental stages. The strong negative effects of *Festuca* competition in the microsite availability treatments observed at harvest support the assumption that the potential performance of de‐novo populations was spatially limited by the experimental units. An effect of genetic diversity depending on the population's origin was only observed for the number of individuals very early in the present experiment and the ratio of generative to vegetative individuals during the second growing season. Invasive de‐novo populations benefitted significantly from increased genetic diversity in Week 4 displaying a higher number of established individuals while native origins were negatively affected in Week 2. In an earlier field experiment within the species' native range, with a subset of source populations used here, we found that invasive transplants performed better compared to native transplants during their vegetative growth phase (Watermann et al. 2022). While this general pattern was confirmed here, the effect of genetic diversity was only detectable in a narrow, easily overlooked time window if not continuously monitored. Therefore, hypothesis I, stating that the effect of increased genetic diversity is more important for invasive origins, can be partially accepted. Such an effect has already been shown for invasive populations compared to native populations as a correlation between standing genetic variation and invasive genotype performance in the field and/or under controlled conditions, for example, for invasive *Phalaris arundinacea* or *Mimulus guttatus* (Lavergne and Molofsky 2007; Zimmer et al. 2023). However, we are only aware of a few studies that have demonstrated this experimentally. In a similar study, Erfmeier et al. (2013) addressed the effect of genetic diversity and propagule pressure in invasive populations of *Senecio vernalis*. Their results showed a life‐stage dependency for effects of genetic diversity and that only initial establishment is positively influenced by genetic diversity. For both their and the present study, it cannot be excluded that measuring direct fitness (seed output, offspring performance) could reveal a lasting effect of increased genetic diversity. Furthermore, in the present experiment, it is likely that the size of experimental units has limited population performance. Even though no apparent signs of stress (e.g., nutrient deficiency) were observed, the number of bolting individuals may have been limited by spatial constraints. Therefore, higher establishment success and performance during the vegetative growth phase might still be ecologically relevant and explain the species' success in the novel range. To elucidate the importance of this advantage during early establishment and whether or not it has a lasting impact, at least one offspring generation should be included in a follow‐up experiment.

### 4.2 Genetic Diversity Does Not Universally Promote Establishment When Microsite Availability Is Limited and the Interactions With Origin Are Highly Context‐Dependent

The importance of high intraspecific diversity is connected to a population's ability to counteract shifting environmental conditions (e.g., Godbout et al. 2020; Smith et al. 2020) and was shown for various environmental constraints as they do occur, for example, along elevational (e.g., Molina‐Montenegro et al. 2011; Watermann et al. 2020) or latitudinal (e.g., Molina‐Montenegro et al. 2018) gradients. Genetic diversity was found to counteract the negative impact of plant density (Cook‐Patton et al. 2017) and increase resistance against disturbance events like grazing (Hughes and Stachowicz 2004). We therefore expected increased genetic diversity to enhance performance in scenarios of low microsite availability where space and thereby safe sites for establishment were constrained due to the presence of competition as realized in our experiment (hypothesis II). Vegetative biomass and abundance at Week 11 were affected by genetic diversity depending on the applied microsite treatment. However, whenever performance was significantly affected by increased genetic diversity, the effect tended to be a negative one. We therefore have to reject hypothesis II that increased genetic diversity generally mitigates the negative effect of restricted microsite availability. Since biological invasions are often associated with bottlenecks, we specifically expected genetic diversity to have a more positive impact with restricted microsite availability for invasive origins (hypothesis III). Over the course of the experiment, very few instances of a significant effect of genetic diversity in relation to microsite availability were observed. If there was a significant interaction, it was negative and it was also more likely to affect native origins.

In the context of biological invasions, genetic diversity is often referred to as explaining the “lag‐phase” that regularly precedes invasive populations' spread after initial establishment (e.g., Suarez and Tsutsui 2008; Lachmuth et al. 2010; Bock et al. 2015). Mechanistically, heterosis by admixture may allow even small founder populations to avoid extinction, as shown for pheasants in the United States (Drake 2006). This effect was also experimentally confirmed for *Mimulus guttatus* (Li et al. 2018). A study on *Plantago lanceolata* furthermore highlighted the importance of additional gene flow for invasive species, as repeated introductions aid populations in overcoming environmental constraints (Smith et al. 2020). Similarly, multiple introductions from different origins and subsequent intraspecific hybridization in *Spartina alterniflora* contributed to its invasive success in China (Hongmei et al. 2019). Populations of invasive *Artemisia ambrosiifolia* exhibited rapid and repeated adaptations (van Boheemen et al. 2019). For invasive *Brachypodium sylvaticum* , rapid adaptation was dated to the time between initial introduction and subsequent spread (Marchini et al. 2018). Our findings corroborate this pattern that genetic diversity can act as a driver of population performance during different stages of the invasion process, but the experimental de‐novo populations did not benefit from this effect in relation to microsite availability. Our findings rather point to genetic diversity being potentially beneficial in relation to other biotic or abiotic factors.

However, as also observed in the present experiment, higher genetic diversity is not unequivocally found to be conducive to invasion success. Local adaptation to the native habitat may lead to maladaptation in novel habitats and admixture may lead to outbreeding depression (Shi et al. 2018). For invasive populations, an experiment with *Centaurea solstitialis* revealed a negative long‐term outcome of admixture (Barker et al. 2019). Furthermore, it is important to keep in mind that most approaches address neutral genetic variation and its actual impact on populations may be overestimated (Teixeira and Huber 2021). For the invasive cane toad *Rhinella marina* , research specifically found an overall reduced genetic diversity but not in loci under selection (Selechnik et al. 2019). Furthermore, for the mite *Tetranychus urticae*, the effect of genetic diversity was specifically shown to be context dependent (Mortier et al. 2021). The authors found that unexpectedly, higher genetic diversity was disadvantageous under challenging environmental conditions. So even while the absolute genetic diversity was comparatively lower in invasive than in native de‐novo populations in our experiment, which might also have dampened a potential positive effect of increasing genetic diversity, it is possible that invasive origins in this species simply do not benefit from increasing genetic diversity. This can be either because increasing genetic diversity increases the potential for maladaptation or because the different levels of genetic diversity do not translate to actual phenotypic differences. Therefore, the effect of genetic diversity in biological invasions is not only species‐ but also highly context‐dependent. This stark context dependency was also found in the present experiment, with a delicate interplay between origin, genetic diversity, microsite availability, and life‐stage.

## 5 Conclusions

The present experiment revealed differences between native and invasive de‐novo populations in establishment and performance. We found differences both in the reaction to increasing genetic diversity as well as in decreasing microsite availability and their interaction that hamper establishment. Although we found instances in which increasing genetic diversity negatively affected the invasive de‐novo populations, the effect was more likely to be a positive one; that is, there is experimental proof that genetic diversity supports the establishment and persistence of invasive populations. In contrast, such promotion through diversity was not observed in native populations, even though the absolute increase in genetic diversity was much larger than in the invasive de‐novo populations. These results highlight the importance of limiting propagule addition also for well‐established invasive species like *J. vulgaris* , supporting bans on import/trade of invasive species, even when they are already considered established or widespread. However, in unfavorable microsite conditions as tested here, genetic diversity did not significantly aid population establishment and was more likely to negatively affect population establishment, especially for native origins. However, whether the biodiversity insurance hypothesis (Loreau et al. 2021; Wright et al. 2021) applies to the availability of safe microsites as an environmental factor of constraint should be tested using more finely tuned levels. Nevertheless, microsite limitation is particularly relevant for the early stages of *J. vulgaris* ' life cycle. To address more sustainable beneficial effects of genetic diversity to counteract environmental constraints that differ between invasive and native populations, we suggest testing other environmental factors such as nutrient shortage or climatic constraints that are limiting growth.

## Author Contributions

**L. Y. Watermann:** conceptualization (equal), data curation (lead), formal analysis (lead), funding acquisition (equal), investigation (lead), methodology (lead), validation (lead), visualization (lead), writing – original draft (lead), writing – review and editing (equal). **W. Durka:** data curation (supporting), formal analysis (equal), funding acquisition (supporting), investigation (supporting), methodology (supporting), supervision (supporting), writing – review and editing (equal). **A. Erfmeier:** conceptualization (lead), formal analysis (supporting), funding acquisition (supporting), methodology (supporting), project administration (lead), supervision (lead), validation (supporting), writing – original draft (supporting), writing – review and editing (equal).

## Conflicts of Interest

The authors declare no conflicts of interest.

## Supporting information


Appendix S1.

## Data Availability

Raw sequence data have been deposited in the European Nucleotide Archive (ENA, https://www.ebi.ac.uk/ena) under project accession number PRJEB56266 (https://www.ebi.ac.uk/ena/browser/view/PRJEB56266). SNP data in vcf format and the SNP filtering R code as well as the raw data from the common garden experiment with the corresponding R code are available via Dryad digital Repository (https://doi.org/10.5061/dryad.0000000dr).
