# Supplementary material for: An Established Plant Invader May Still Benefit From Increasing Genetic Diversity—Insights From Artificial Populations in a Common Garden Experiment
Source: Ecol Evol. 2025 Feb 14;15(2):e70963. doi: 10.1002/ece3.70963 (PMC11826087; doi:10.1002/ece3.70963)
Supplement: Supplementary file 1 — Appendix S1. [file ECE3-15-e70963-s001.docx]

Appendix

Table A1: Source populations

**Table A1: Origin of source populations.**

Figure A2: Map of Source populations
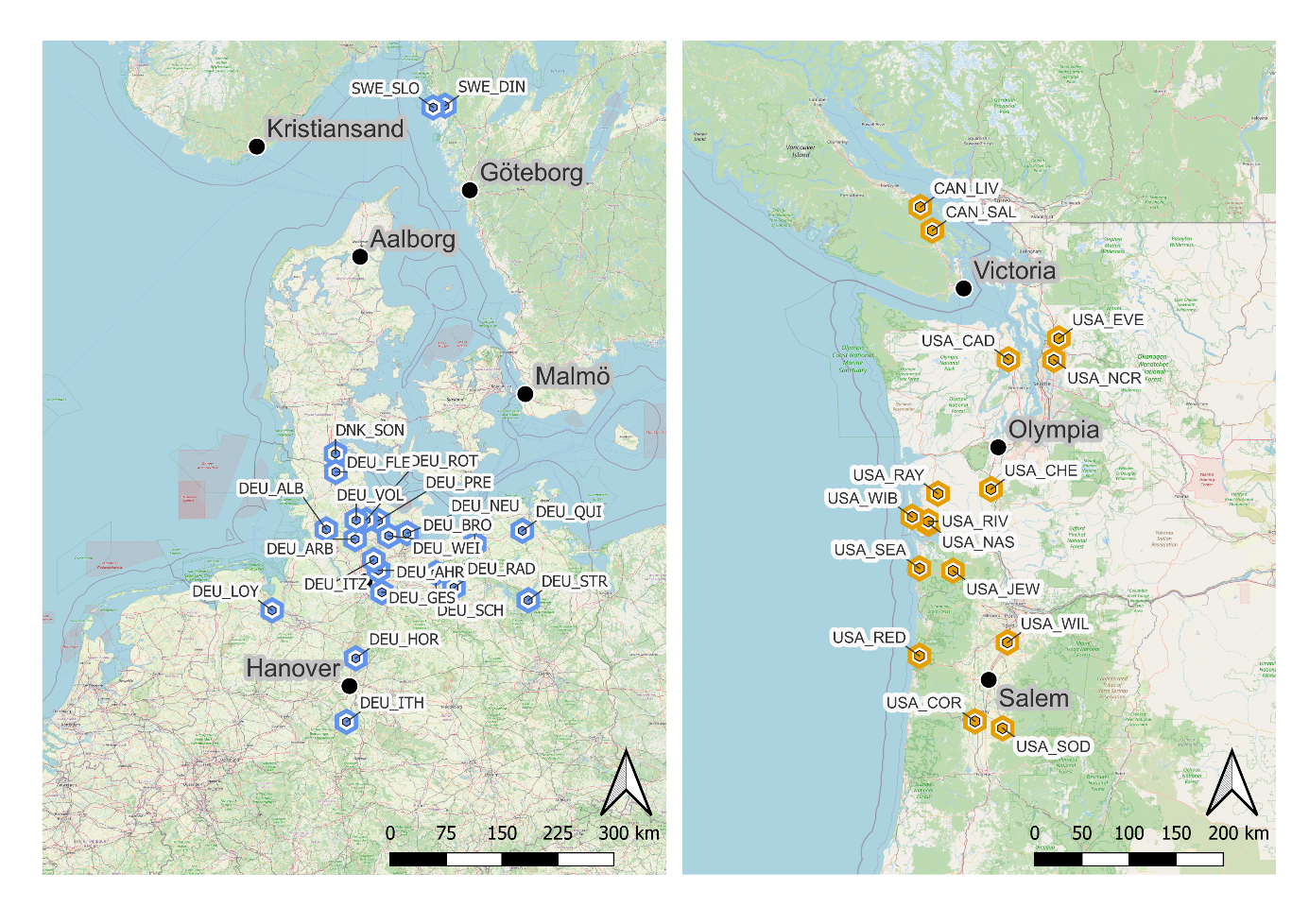


**Figure A2: Location of the source populations used for seed sampling.** Blue symbols indicate the native populations, orange symbols show invasive populations. For detailed information about the location of the populations see Table A1. Map was created using QGIS (QGIS Development Team 2024). QGIS Geographic Information System. Open Source Geospatial Foundation Project. http://qgis.osgeo.org. Map data © OpenStreetMap contributors and available from https://www.openstreetmap.org


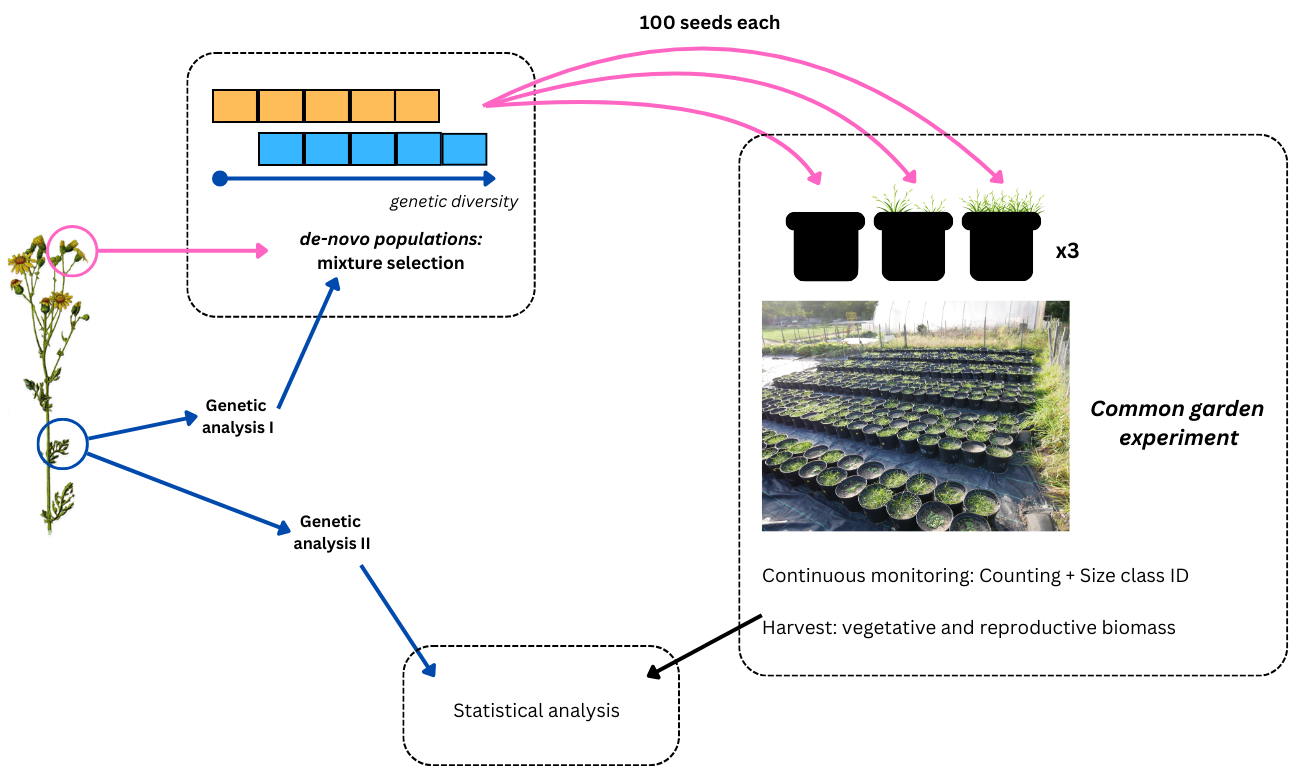
Figure A3: Methodological approach.

**Figure A3: Overview of the methodological approach.**

The image depicts an overview of the experimental set-up leading up to the statistical analyses. Pink arrows signify usage of seed material for the creation of *de-novo populations*, while blue arrows signify usage of leaf material for genetic analysis. Orange indicates invasive origin and blue native origin. Since overall genetic diversity was lower in invasive origins, the invasive *de-novo populations* occupied a uniquely low range for genetic diversity, while native *de-novo* populations occupied a uniquely high range for genetic diversity. Leaf material was used for genetic analysis twice. The first analysis was used to select the experimental *de-novo populations* while the second analysis (after finetuning the steps) was used for final statistical analysis. The containers depict the experimental units with their 3 levels of microsite availability. Each (*de-novo*) population x microsite availability combination was repeated three times.

A4: Genetic analysis

We adopted a population pool approach for the analysis of genomic variation. Thus, for each source population, we pooled 1 mg of leaf material of each of the 20 maternal individuals to produce a population sample. DNA was extracted using the peqGold Plant DNA mini kit (VWR International, USA) following the manufacturers’ protocol. DNA content per sample was assessed using a Qubit Fluorometer (Thermo Fischer Scientific Inc., USA) and subsequently normalized to 20ng/µl. We adopted a reduced representation sequencing approach for single-nucleotide polymorphism (SNP) detection and followed the ddRAD protocol of Peterson et al. ( 2012) for library preparation. Therefore, for each population pool sample, 200ng of genomic DNA were digested with restriction enzymes EcoRI and Mspl and subsequently adapters we ligated to the fragments. Adapters contained one of 48 unique 5bp-barcodes. Each set of samples containing one of the 48 unique barcodes was then pooled and cleaned with the Promega-Wizard Gel and PCR Clean-up Kit (Promega GmbH, Germany). We selected fragments between 350 – 450bp (Pippin-prep, Sage science, USA). PCR amplification was then conducted in 24 parallel reactions per pool for 12 cycles with Illumina sequencing primers with a 6bp index. Subsequently, the samples were again cleaned using the Wizard Gel and PCR Clean-up kit and additionally with AMPure XP beads (Beckman Coulter, USA). The resulting libraries were analyzed with an Agilent 2100 Bioanalyzer (Agilent technologies, USA) for quality check. Finally, we equimolarly combined libraries. Pair-end sequencing was done on an Illumina HiSeq 2000 with the Cluster Generation Kit (PairedEnd, v3-HS Kit; 10% PhiX v3) and the SBS Sequencing Kit (200 cycles, v3-HS). Raw sequence data have been deposited in the European Nucleotide Archive (ENA, https://www.ebi.ac.uk/ena) under project accession number PRJEB56266 ([https://www.ebi.ac.uk/ena/browser/view/PRJEB56266](https://www.ebi.ac.uk/ena/browser/view/PRJEBxxxx)). SNP data in vcf format and the SNP filtering R code will be made available via Dryad digital Repository.

Figure A5: *F_st_* table

*F_ST_* Table for native (left) and invasive (right) source populations

Figure A6: *F_st_* and geographic distance


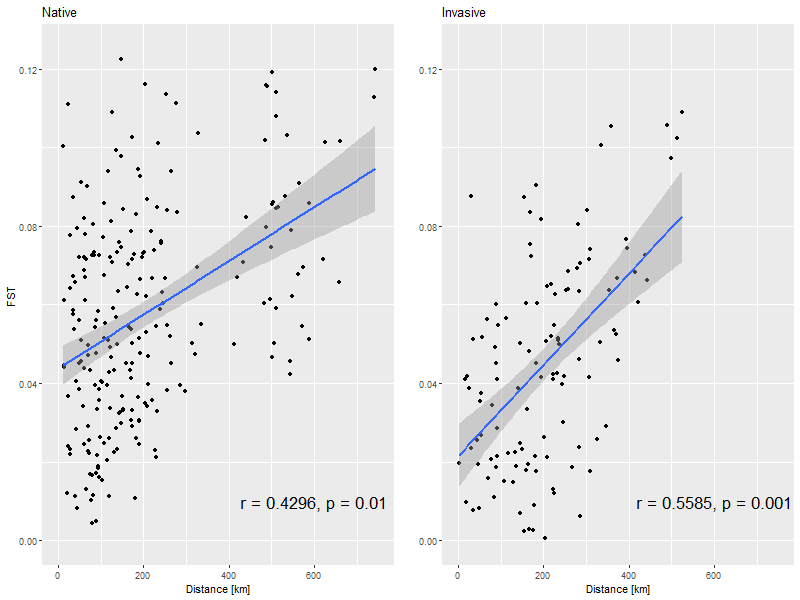


**Figure A6: Relationship of among-source population *F_ST_* and their geographic distance from each other.** Native source populations are on the left. The group of data points in the upper right are related to the Swedish source populations. For native, but without Swedish source populations, r = 0.2037 and p = 0.135. Invasive source populations on the right.

Figure A7: Mixture selection


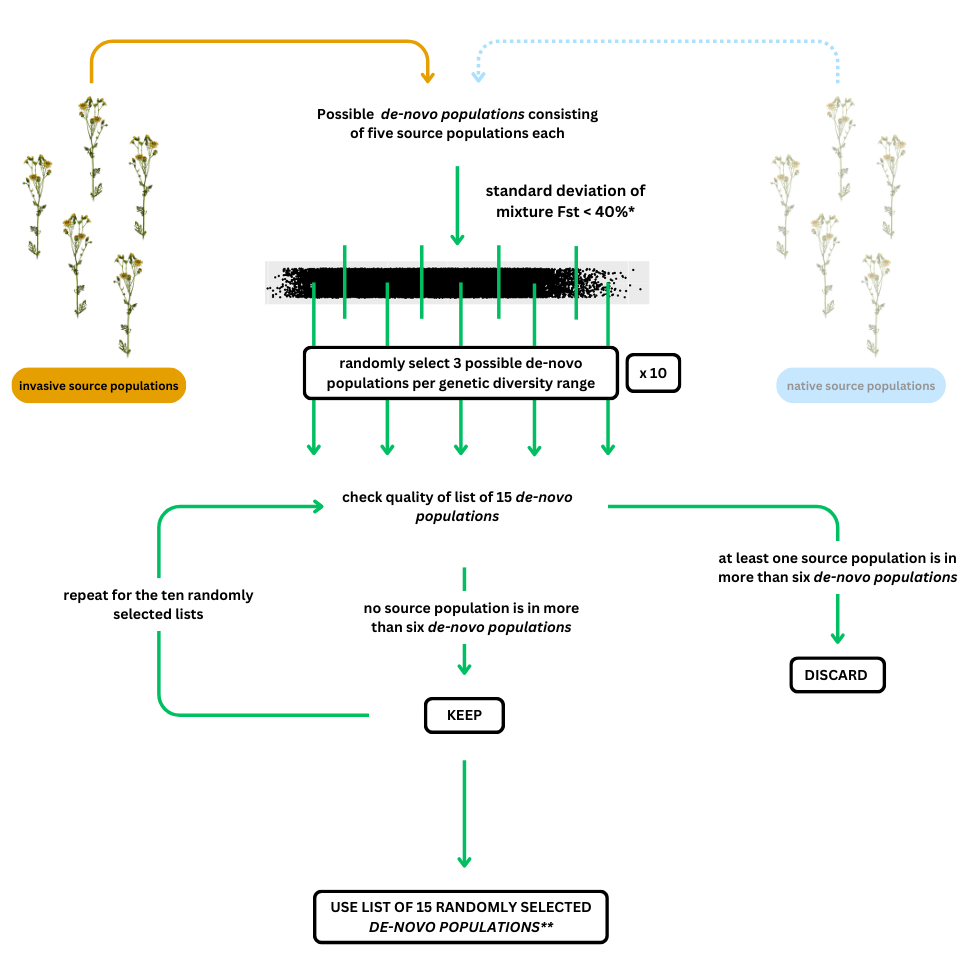


**Figure A7: Detailed depiction of processes used to select *de-novo populations.*** Note that the selection of *de-novo populations* was done for the two origins separately and there were no *de-novo populations* that consisted of a mixture between the origins. After calculation all possible combinations within each range (5 out of 16 for invasive and 5 out of 22 for native origins), mixtures were the mean mixtures *F_st_* exhibited a standard deviation of more than 40% were removed (*except for invasive de-novo populations with a mean mixture *F_st_*  <0.0224). The entire range of genetic diversity was divided into six equal bands, however, only five per origin, as each one band was exclusive to one of the origins (*F_s_*_t_ <0.0224 for invasive origins, *F_ST_* > 0.1249 for native origins). Per range, three *de-novo populations* were randomly chosen and combined in one list. This step was always repeated 10 times. Starting with the first list, each list was checked for equal incorporation of source populations (maximum of six times per list). Only lists where this criterion was met were kept for potential use in the common garden experiment. **Of all kept lists, the one with the least amount of source population repeats (i.e. number of times a source population was in one of the fifteen *de-novo populations*) was used.

A8: Mixture selection

For our experimental *de-novo populations* we created seed mixtures of 5 source populations each at different levels of genetic variation. Genetic variation of the *de-novo populations* was predicted based on the mean pairwise *F*_ST_ values among the five source populations. Pairwise *F*_ST_ values quantify the proportion of genetic variation, i.e., genetic differentiation, among populations. To select source populations to be mixed, we first built all possible combinations for five populations within region (5 out of 24 for native and 5 out of 16 for invasive origin), resulting in 42,504 and 4,368 possible combinations respectively. For each of these combinations we calculated the mean pairwise F_ST_-value (mean F_ST_ of the 10 pairwise F_ST_-values of 5 populations) and its standard deviation. The mean *F*_ST_ ranged from 0.0224 to 0.1801 for native and from 0.0 to 0.1249 for invasive mixtures. Consequently, the range *F*_ST_ < 0.0224 was exclusive to invasive while the range *F*_ST_ > 0.1249 was exclusive to native *de-novo* populations. For each origin, we selected a total of 15 *de-novo populations*, with each three representing the origin-exclusive range. We excluded all combinations where the standard deviation was larger than 40% of the mean, except for invasive exclusive range *F*_ST_ < 0.0224 which had very low *F*_ST_ values. This assured that only combinations consisting of reasonably similar or dissimilar source populations were included. This allowed us to retain enough combinations for random selection of *de-novo populations* including all source populations. In a next step, the shared range 0.0224 < *F*_ST_ < 0.1249 was divided equally in four parts. For each origin, the origin-exclusive range was added, thus resulting in five mean F_ST_ groups.

Within each of the five sections of the total range of mean *F*_ST_ values, we randomly selected three *de-novo populations* to obtain a total of 15 populations for use in the common garden experiment. Those *de-novo populations* were thus equally distributed along the entire genetic diversity gradient for each range of origin. For each origin we generated 10 random selections of 15 populations. Of these ten, we choose the list in which the original source populations were most evenly distributed in the artificial *de-novo populations,* and that also incorporated any given source population no more than six times. If no list qualified the last criterion, we generated 10 additional lists and repeated the selection process. We always used all ten lists, therefore ending up with the best of ten (times the rounds needed) that incorporated no source population more than six times. Ultimately, the diversity levels of the artificial *de-novo populations* ranged from mean *F*_ST_ 0.036 to 0.1434 for native and from 0.015 to 0.1129 for invasive origins.
